# Prognostic Value of Neutrophil-to-Lymphocyte Ratio in Stroke: A Systematic Review and Meta-Analysis

**DOI:** 10.3389/fneur.2021.686983

**Published:** 2021-09-24

**Authors:** Wenxia Li, Miaomiao Hou, Zhibin Ding, Xiaolei Liu, Yuan Shao, Xinyi Li

**Affiliations:** ^1^Shanxi Academy of Medical Sciences, Shanxi Bethune Hospital, Tongji Shanxi Hospital, Third Hospital of Shanxi Medical University, Taiyuan, China; ^2^Shanxi Medical University, Taiyuan, China

**Keywords:** inflammation, ischemic stroke, hemorrhagic stroke, meta-analysis, neutrophil-to-lymphocyte ratio, prognosis, systematic review

## Abstract

**Background:** Stroke has become a major problem around the world, which is one of the main causes of long-term disability. Therefore, it is important to seek a biomarker to predict the prognosis of patients with stroke. This meta-analysis aims to clarify the relationship between the neutrophil-to-lymphocyte ratio (NLR) and the prognosis of stroke patients.

**Methods:** This study was pre-registered in PROSPERO (CRD42020186544). We performed systematic research in PubMed, Web of Science, and EMBASE databases for studies investigating the prognostic value of NLR. Based on the enrolled studies, patients were divided into the low-NLR cohort and the high-NLR cohort. Odds ratios (ORs) with 95% confidence intervals (CIs) were extracted and analyzed by the Review Manager 5.3 and Stata 12.0 software. Heterogeneity was estimated by using Cochran's *Q* test and *I*^2^ value. Sensitivity analyses and subgroup analyses were also performed to explore the potential sources of heterogeneity. Publication bias was assessed with funnel plots and assessed by Egger's tests.

**Results:** Forty-one studies with 27,124 patients were included. In the overall analysis, elevated NLR was associated with an increased mortality in acute ischemic stroke (AIS) patients (OR = 1.12, 95% CI = 1.07–1.16) and in acute hemorrhagic stroke (AHS) patients (OR = 1.23, 95% CI = 1.09–1.39), poorer outcomes in AIS patients (OR = 1.29, 95% CI = 1.16–1.44), and in AHS patients (OR = 1.11, 95% CI = 1.03–1.20). While in terms of hemorrhagic transformation (HT), elevated NLR was associated with an increased incidence of HT in AIS patients (OR = 1.15, 95% CI = 1.08–1.23).

**Conclusions:** This study demonstrated that elevated NLR was significantly associated with poor prognosis of stroke patients. High NLR is associated with a 1.1- to 1.3-fold increased risk of poor outcomes of AIS/AHS patients. NLR could be helpful as a potential prognostic biomarker to guide clinical decision making.

**Systematic Review Registration:** https://www.crd.york.ac.uk/prospero/display_record.php?ID=CRD42020186544.

## Introduction

With almost 6 million deaths and more than 10% of all mortality every year, stroke has become one of the predominant threats to human health (1). There are two types of strokes, one is ischemic stroke, which accounts for 85% of all acute stroke, and the other is hemorrhagic stroke. According to previous reports, about 40% of all stroke deaths are attributable to hemorrhagic stroke (2). Currently, the major treatment for acute ischemic stroke is reperfusion therapy, which includes intravenous tissue plasminogen activator and endovascular therapy (EVT) (3). Exploring the key factors that affect the prognosis of stroke patients is crucial for clinicians to design appropriate treatments to improve the clinical efficacy and prognosis to stroke patients.

As we all know, there are two important pathophysiological mechanisms of stroke including oxidative stress and inflammation. After stroke, the inflammatory response is activated and plays a significant role in secondary brain injury (4). In recent years, the immunity has emerged as a new breakthrough target in the treatment strategy for acute stroke. Meanwhile, it is non-displaceable in predicting a poor prognosis (5). However, it is a complex process that can induce the activation and immunosuppression of a variety of inflammatory cells. Previous studies have found the different roles of neutrophils and lymphocytes in the progression and prognosis after stroke. Neutrophils could re-infiltrate the ischemic site in the first few hours after stroke, and then release chemical mediators related to increased tissue damage and poor neurological prognosis (6). At the same time, stroke could trigger a special immunosuppressive state (4), such as the activation of neutrophils, which leads to a decrease in lymphocytes (7), and certain types of lymphocytes are considered to be important brain protective immune regulators; the decrease of these lymphocytes may lead to deterioration of nerve function (8). Recently, the neutrophil-to-lymphocyte ratio (NLR) has become a powerful predictor of death in patients with cardiovascular disease or peripheral arterial occlusive disease. Previous studies reported a correlation between stroke severity and NLR determined at admission. Several studies suggested that the initial NLR was associated with mortality and infarct size in ischemic stroke patients.

However, the value of NLR in predicting the poor prognosis of stroke patients is still controversial. Some studies showed that NLR had no obvious effect on mortality (9, 10), while some studies demonstrated that a high NLR was an independent predictor of poor clinical outcomes in patients with stroke (11, 12). Thus, the aim of this study was to perform a meta-analysis to clear the relationship between NLR and the prognosis in patients with stroke.

## Materials and Methods

### Search Strategy

Registered in PROSPERO with the number CRD42020186544, this meta-analysis searched the databases, including PubMed, Web of Science, EMBASE, Scopus, and Google Scholar, which were papers published from the time of inception of the database to January 2021. We used the following search terms: “NLR or neutrophil-to-lymphocyte ratio or neutrophil-lymphocyte ratio” and “stroke or acute ischemic stroke or cerebrovascular accident or CVA or AIS or TIA or intra-cerebral hemorrhage or intracranial hemorrhage or AHS or subarachnoid hemorrhage” (Supplementary Table 1). Two investigators independently performed the literature search and resolved any disagreements via discussion. We screened retrieved articles in citation lists manually to ensure sensitivity of the search strategy.

### Inclusion and Exclusion Criteria

The following eligibility criteria were utilized to reduce clinical heterogeneity: (a) patients were diagnosed with acute stroke, including ischemic stroke, and hemorrhagic stroke; (b) on or after admission, white blood cell counts and NLR were assessed or can be calculated; (c) odds ratios (ORs) or risk ratios (RRs) were provided with 95% confidence interval (CI) for survival outcomes or functional outcomes; and (d) prospective or retrospective cohort studies were considered eligible. Exclusion criteria were (a) the article was conference abstracts, letters, case reports, reviews, unrelated articles; (b) patients with systematic inflammatory disorders, such as recent myocardial infarction, liver or kidney failure, history of cancer; (c) the end point event of the study was not death, disabled, or hemorrhagic transformation; and (d) studies without enough data (refers to the absence of odds ratio or related data used to estimate odds ratio, lack of neutrophil–lymphocyte ratio or functional outcome after discharge). Disagreements were resolved by consensus between the two investigators.

### Data Extraction

Relevant data were extracted by two independent investigators (WL and MH) from the eligible studies, including patients' characteristics, clinical data, and laboratory data such as first author, year of publication, patients area, sample size, study period, mean or median age, gender, National Institutes of Health Stroke Scale (NIHSS) or Glass Coma Scale (GCS), stroke type, time of onset, comorbid status, initial treatment, sampling time of the blood, research method, and cutoff value of NLR. We collected OR and 95% CI on the mortality (short term or long term), functional outcomes, and symptomatic intracranial hemorrhage, or parenchymal hematoma. We used multivariate regression analysis data, if the ORs of univariate and multivariate regression analyses were both available in the study. Any disagreement was settled via discussion with a third investigator.

### Outcomes

The functional status was characterized by modified Rankin Scale (mRS) during clinical follow-up, with poor functional outcome as mRS ≥ 3, whereas the survival outcomes were measured by the occurrence of spontaneous intracerebral hemorrhage (sICH) or hemorrhagic transformation (HT), and mortality.

### Quality Assessment

We applied the Newcastle–Ottawa Scale (NOS), which includes three factors: selection, comparability, and exposure to assess the quality of each enrolled study. The total score ranged from 0 to 9, and the score of 3 or less, 4–6, or 7 or more were considered to have low, intermediate, or high quality, respectively.

### Statistical Analyses

The Review Manager version 5.3 software from Cochrane was applied in the analysis, and we utilized STATA 12.0 (STATA Corporation, College Station, TX, USA) to evaluate publication bias generated in the study. The prognostic value of NLR in stroke patients was estimated by forest and funnel plots. Based on the enrolled studies, patients were divided into the low-NLR cohort and the high-NLR cohort according to different cutoff values. Due to large sample size, we assumed that an OR is a good approximation to RR in our study; so we use the OR as the effect size of this meta-analysis. The log (OR) and its standard error were calculated by OR and 95% CI and used for aggregation. We merged the OR and 95% CI to analyze the implication of the NLR with poor endpoints. Furthermore, the heterogeneity was assessed comprehensively through subgroup analysis and sensitivity analysis. Heterogeneity between the studies was evaluated by Cochran's *Q* test and *I*^2^ statistic. A random-effect model was applied to calculate the pooled ORs and 95% CIs if there was significant heterogeneity among the enrolled studies (*I*^2^ > 50%, or *p* < 0.10); otherwise, the fixed-effect model was adopted (*I*^2^ <50% or *p* > 0.10). Publication bias was assessed by Egger's test. A *p*-value <0.05 was considered significant statistically.

## Results

### Literature Research

**Figure 1:**
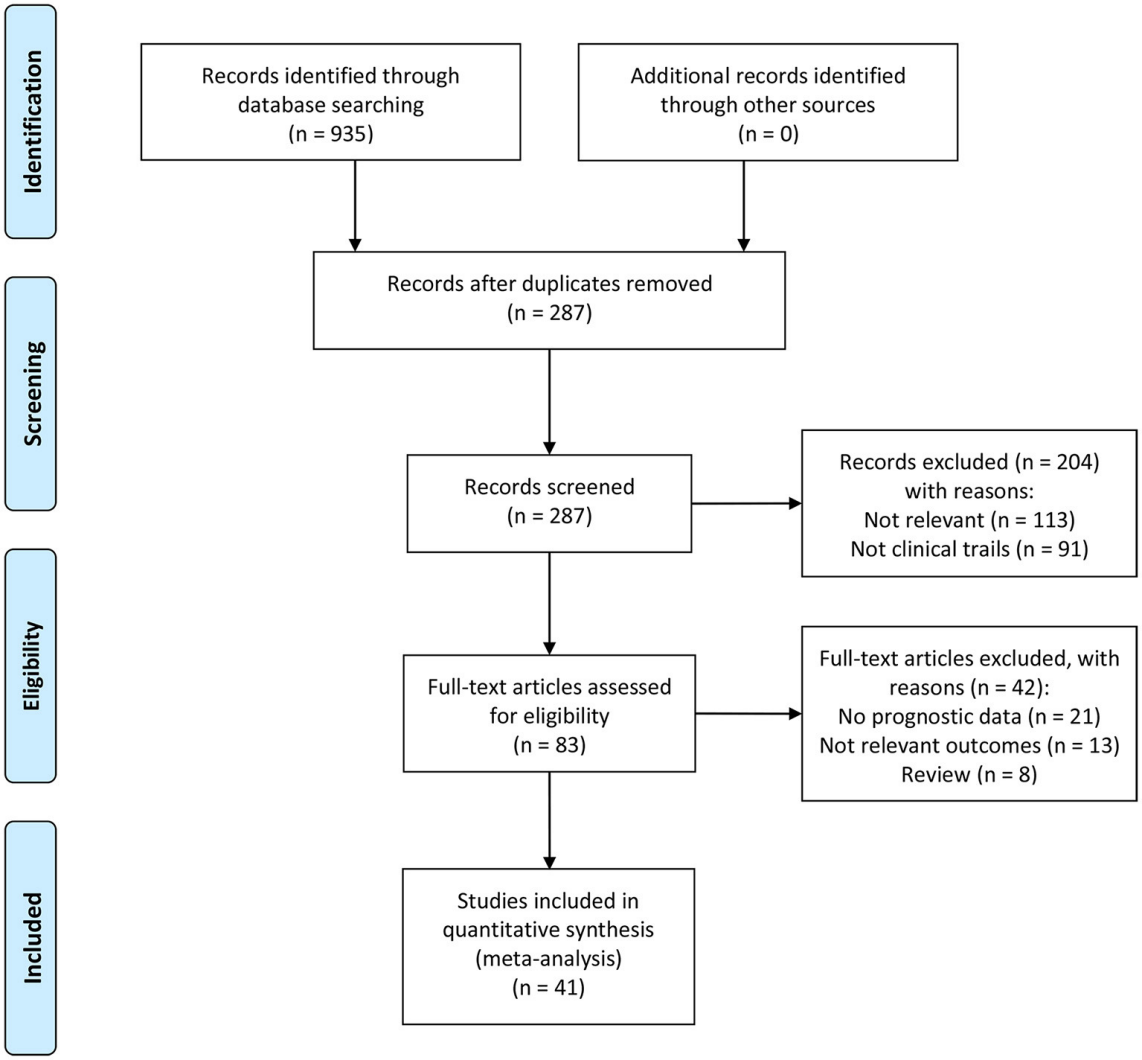
The flowchart of the selection process of studies according to the Preferred Reporting Items for Systematic Reviews and Meta-Analyses guidelines (PRISMA).

Figure 1 shows the research flow diagram. A total of about 935 potentially relevant records were selected after the initial literature research. After removing the duplications, a total of 287 studies were reviewed by titles and abstract. Of the remaining 287 articles, 204 papers were excluded due to meeting the exclusion criteria. Then, we inspected the remaining 83 articles with full texts, in which 42 studies lack enough data. Eventually, 41 articles with 27,124 patients were included in our analysis.

### Study Characteristics

The clinical characteristics of patients described in the studies are shown in Table 1. Characteristics of the included studies are reflected in Table 2. Of the 41 studies, 30 reported ischemic stroke events (9–36, 48, 49), and 11 reported hemorrhagic stroke events (37–47). Nine studies were prospective in design, and 32 studies were retrospective. Among them, blood samples were taken out on admission, in 24 h after admission, in 48 h after admission, or in the first week after admission. For ischemic stroke, the sample size ranges from 51 to 3,013, and the research regions cover Asia, Europe, Australia, and North America. Most of the studies contain multiple stroke types, including large artery atherosclerosis (LAA) type, cardioembolism (CE) type, small vessel occlusion (SVO) type, stroke of other determined etiology (SOE) type, and stroke of undetermined etiology (SUE) type. In terms of hemorrhagic stroke, six of them were from China, and four were from Europe. The most frequently evaluated subtype of hemorrhagic stroke was ICH (*n* = 9) and subarachnoid hemorrhage (*n* = 2). The cutoff values of NLR varied between studies. Overall, all ORs and 95% CI are adjusted and obtained from the multiple regression analysis. The NOS scores ranged from 6 to 9, indicating a moderate to high quality of included studies.

**Table 1:** Clinical characteristics of patients described in the studies.

**Author and year**	**Age**	**Gender (M/F)**	**Severity**	**Stroke type**	**CAD**	**HBP**	**DM**	**Smoking**	**Therapy**
**Ischemic stroke**									
Zhang 2020 (11)	Mean = 73 ± 13	1,801/1,212	NIHSS = 4.65 ± 6.07	CE/LAA/SAO	335/3,013	2,175/3,013	1,065/3,013	998/3.013	NA
Zhang 2020 (13)	NA	285/113	NA	LAA	Non	148/398	57/398	230/398	NA
Ying 2020 (14)	Mean = 67	128/80	NA	AIS	Non	165/208	56/208	74/208	IVT
Switonska 2020 (15)	IQR = 67 [55–78]	22/29	NIHSS = 11 (6–16)	LAA/SVO/ramyaCE/SOE/SUE	13/51	38/51	16/51	13/51	IVT, IVT + MT, MT
Semerano 2020 (12)	IQR = 74.2 (65.7–79.7)	297/213	NIHSS = 6 (3–14)	LAA/SVO/ramyaCE/SOE/SUE	95/510	352/510	95/510	135/510	IVT 38.4%
Cao 2020 (16)	Mean = 66.81 ± 12.58	451/182	NIHSS = 3 (2–6)	TACI/PACI/ramyaPOCI/LACI	87/633	515/633	262/633	Non	IVT 2%
Wang 2019 (17)	Mean = 63.9 ± 13.7	479/329	NA	LAA/SVO/ramyaCE/SOE/SUE	45/808	502/808	202/808	245/808	Antiplatelet/anticoagulant
Sun 2019 (18)	IQR = 61 (53–71)	106/52	NA	LHI	Non	Non	Non	80/158	IVT/MT 24.1%
Semerano 2019 (19)	IQR = 71 (61–80)	226/207	NIHSS = 17 (11–21)	LAA/CE/ramyaSOE/SUE	57/433	263/433	90/433	101/433	MT
Nam 2019 (20)	IQR = 69 (60–76)	209/140	NIHSS = 3 (2–6)	LAA	Non	214/349	128/349	149/349	IVT/MT 10.6%
Kozyolkin 2019 (21)	IQR = 74 (65–78)	71/65	NIHSS = 12 (10–14)	RCIHS	Non	Non	31/136	Non	NA
Kocaturk 2019 (22)	Mean = 67	57/50	NIHSS = 10 (10–15)	LAA/SVO/ramyaCE/SOE/SUE	Non	67/107	33/107	Non	IVT/MT 21.4%
Lim 2019 (23)	NA	59/45	NA	LAA/SVO/ramyaCE/SOE/SUE	13/104	72/104	32/104	25/104	NA
Malhotra 2018 (24)	Mean = 64.3 ± 14.4	333/324	NIHSS = 7 (4–13)	AIS	153/657	509/657	225/657	219/657	IVT
Pikija 2018 (25)	IQR = 74 (60–81)	86/101	NIHSS = 18 (13–22)	LAA/CE/ramyaSOE/SUE	Non	119/187	24/187	Non	EVT
Shi 2018 (10)	Mean = 64	242/130	NA	AIS	Non	288/372	77/372	146/372	IVT
Wang 2018 (26)	IQR = 65 (57–73)	200/132	NIHSS = 16 (13–21)	LAA/CE	84/332	220/332	66/332	Non	EVT
Yu 2018 (27)	Mean = 70.0 ± 16.0	253/201	SSS = 54 (44–56)	AIS	Non	256/454	90/454	54/454	Except for IVT
Yilmaz 2017 (9)	IQR = 50 (13–96)	53/53	NA	AIS	Non	7/106	Non	Non	Antiplatelet/anticoagulant
Xue 2017 (28)	Mean = 61.8 ± 10.2	185/107	NA	LAA/SVO/ramyaCE/SOE/SUE	Non	223/292	97/292	110/292	Antiplatelet/anticoagulant
Qun 2017 (29)	IQR = 70	80/63	NIHSS = 6(5–7)	AIS	Non	99/143	30/143	19/143	Antiplatelet
Fan 2017 (30)	IQR = 63 (52–76)	216/146	NIHSS = 9 (5–13)	AIS	47/362	292/362	50/362	Non	NA
Fang 2017 (31)	NA	1,092/639	NA	AIS	90/1,731	1,293/1,731	705/1,731	443/1,731	Antiplatelet
Guo 2016 (32)	NA	123/66	NA	AIS	23/189	122/189	57/189	62/189	IVT
Maestrini 2015 (33)	IQR = 71 (60–80)	430/416	NIHSS = 10 (6–16)	AIS	Non	519/846	129/846	Non	IVT
Brooks 2014 (34)	Mean = 67	54/62	NIHSS = 17 (148), mRS = 4 (0–6)	AIS	Non	Non	Non	Non	EVT
Tokgoz 2014 (35)	Mean = 69.37 ± 13.96	81/70	NA	AIS	33/151	80/151	46/151	44/151	Antiplatelet/anticoagulant
Tokgoz 2013 (36)	Mean = 69.37 ± 13.96	125/130	NA	AIS	61/255	147/255	72/255	69/255	Antiplatelet/anticoagulant
**Hemorrhagic stroke**									
Qin 2019 (37)	IQR = 50 (46–55)	157/56	NIHSS = 10 (5–12.3), GCS = 13 (7–15)	sICH	0.042	0.728	0.094	0.338	NA
Giede-Jeppe 2019 (38)	NA	98/221	NA	SAH	Non	183/319	Non	Non	NA
Lattanzi 2018 (39)	Mean = 66.7 ± 12.4	76/132	NIHSS = 9 (6–14)	ICH	24/208	129/208	44/208	39/208	NA
Qi 2018 (40)	IQR = 57.6 (28.0–79.0)	368/190	NA	ICH	Non	Non	126/558	132/558	NA
Tao 2017 (41)	Mean = 58.5 ± 13.0	216/120	GCS = 11 (7–13)	ICH	Non	189/336	10/336	81/336	NA
Sun 2017 (42)	Mean = 64.2 ± 13.8	234/118	NA	ICH	Non	290/352	43/352	71/352	NA
Giede-Jeppe 2017 (43)	NA	457/398	NA	ICH	Non	705/855	228/855	278/855	NA
Lattanzi 2017 (44)	Mean = 66.9 ± 12.5	123/69	NIHSS = 9 (6–14)	ICH	23/192	123/192	40/192	39/192	NA
Lattanzi 2016 (45)	Mean = 67.1 ± 12.51	63/114	NIHSS = 9 (6–14)	ICH	23/177	116/177	39/177	36/177	NA
Wang 2016 (46)	Mean = 67.97 ± 13.75	141/83	GCS = 12.64 ± 3.49	ICH	Non	166/224	19/224	Non	NA
Tao 2016 (47)	Mean = 55.9 ± 11.9	88/159	NA	SAH	Non	94/247	25/247	51/247	NA

*LAA, large artery atherosclerosis; CE, cardio embolism; SVO, small vessel occlusion; SUC, stroke of undetermined cause; SOE, stroke of other determined etiology; TACI, total anterior cerebral infarction; PACI, partial anterior cerebral infarction; POCI, posterior cerebral infarction; LACI, lacunar cerebral infarction; AIS, acute ischemic stroke; sICH, spontaneous intracerebral hemorrhage; SAH, subarachnoid hemorrhage; LHI, large hemispheric infarction; RCIHS, recurrent cerebral ischemic hemispheric stroke; CAD, coronary artery disease; HBP, high blood pressure; DM, diabetes mellitus; IVT, intravenous thrombolysis; MT, mechanical thrombectomy; EVT, endovascular therapy; GCS, Glasgow Coma Score; NIHSS, National Institutes of Health Stroke Scale; SSS, Scandinavian Stroke Scale; IQR, interquartile range; NA, not available; Non, none*.

**Table 2:** Characteristics of the studies included in the meta-analysis.

**Author and year**	**Period**	**Region**	**Sample size**	**Sample time**	**Study type**	**NOS**
**Ischemic stroke**						
Zhang 2020 (11)	2016–2018	China	3,013	On admission	Retrospective	8
Zhang 2020 (13)	2012–2018	China	398	24 h	Prospective	9
Ying 2020 (14)	2016–2018	China	208	On admission/24 h/7 days	Prospective	8
Switonska 2020 (15)	2017–2018	Poland	51	On admission	Retrospective	7
Semerano 2020 (12)	2008–2015	Italy	510	48 h	Retrospective	7
Cao 2020 (16)	2017–2018	China	633	24 h	Retrospective	7
Wang 2019 (17)	2014–2015	China	808	24 h	Retrospective	6
Sun 2019 (18)	2016–2019	China	158	48 hH	Retrospective	8
Semerano 2019 (19)	2008–2017	Barcelona	433	On admission/24 h	Retrospective	8
Nam 2019 (20)	2010–2015	Korea	349	24 h	Retrospective	7
Kozyolkin 2019 (21)	NA	Ukraine	136	On admission	Prospective	9
Kocaturk 2019 (22)	2017–2018	Turkey	107	24 h	Retrospective	8
Lim 2019 (23)	2015–2017	Korea	104	On admission	Prospective	8
Malhotra 2018 (24)	2011–2015	US	657	On admission	Retrospective	8
Pikija 2018 (25)	2012–2016	Australia	187	On admission	Retrospective	7
Shi 2018 (10)	NA	China	372	On admission	Prospective	8
Wang 2018 (26)	2014–2016	China	332	On admission	Retrospective	8
Yu 2018 (27)	2009–2013	Australia	454	On admission	Retrospective	7
Yilmaz 2017 (9)	2000–2014	Turkey	106	24 h	Retrospective	7
Xue 2017 (28)	2014–2015	China	292	24 h	NA	8
Qun 2017 (29)	2015–2016	China	143	24 h	NA	7
Fan 2017 (30)	2014–2015	China	362	On admission	Retrospective	6
Fang 2017 (31)	2012–2014	Taiwan	1,731	On admission	Retrospective	8
Guo 2016 (32)	2012–2015	China	189	On admission	Prospective	7
Maestrini 2015 (33)	NA	France, Finland	846	On admission	Prospective	6
Brooks 2014 (34)	2008–2011	US	116	On admission	Retrospective	8
Tokgoz 2014 (35)	2007–2013	Turkey	151	On admission	Retrospective	7
Tokgoz 2013 (36)	2007–2012	Turkey	255	On admission	Retrospective	8
**Hemorrhagic stroke**						
Qin 2019 (37)	2017–2018	China	213	On admission	Retrospective	7
Giede-Jeppe 2019 (38)	2008–2012	Germany	319	On admission	Prospective	8
Lattanzi 2018 (39)	2008–2017	Italy	208	24 h	Retrospective	8
Qi 2018 (40)	2010–2017	China	558	On admission	Retrospective	6
Tao 2017 (41)	2010–2013	China	336	On admission	Retrospective	7
Sun 2017 (42)	2011–2014	China	352	24 h	Retrospective	8
Giede-Jeppe 2017 (43)	2006–2014	Germany	855	On admission	Retrospective	8
Lattanzi 2017 (44)	2008–2016	Italy	192	On admission	Retrospective	7
Lattanzi 2016 (45)	2008–2015	Italy	177	On admission	Retrospective	8
Wang 2016 (46)	2012–2014	China	224	On admission	NA	7
Tao 2016 (47)	2014–2015	China	247	24 h	Prospective	7

*NOS, Newcastle–Ottawa Scale; NA, not available*.

### Overall Prognostic Analysis

#### Association of Neutrophil-Lymphocyte Ratio and Mortality

**Figure 2:**
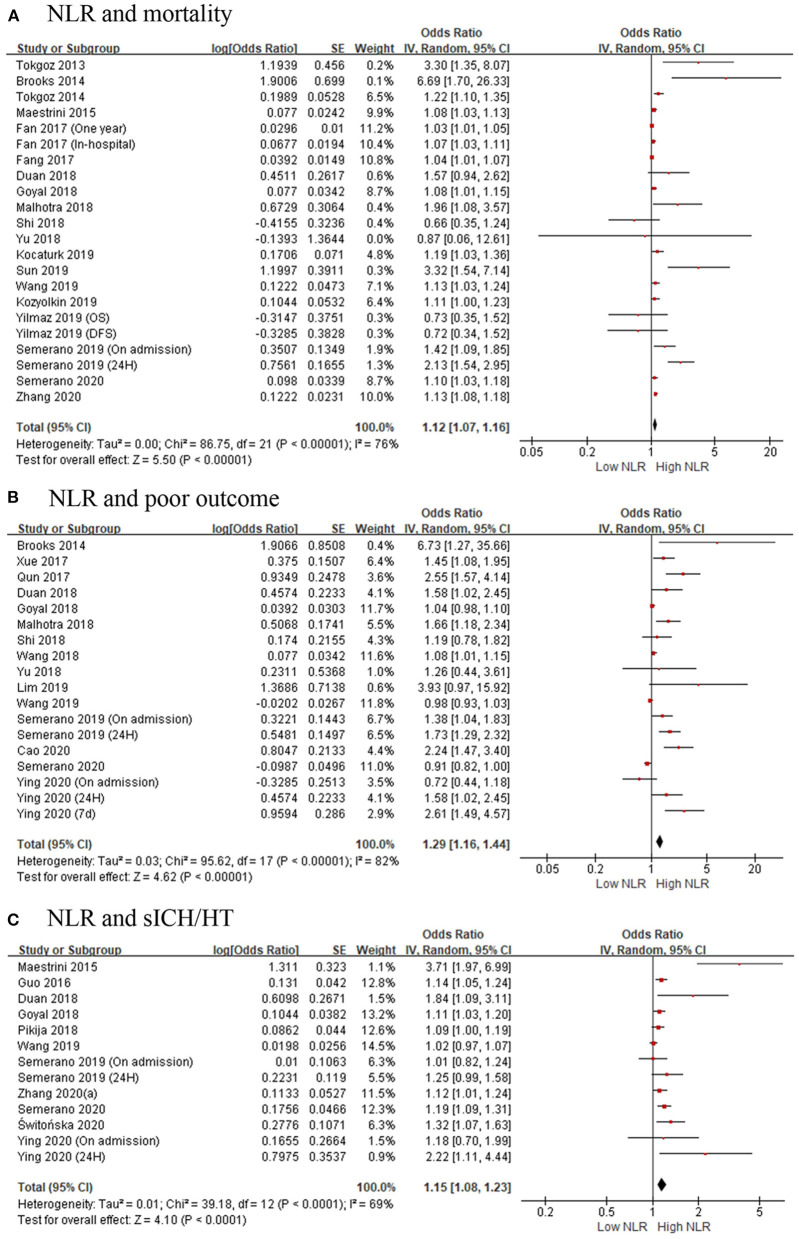
Forest plots of included studies evaluating the association in ischemic stroke patients between **(A)** neutrophil-to-lymphocyte ratio (NLR) and mortality, **(B)** NLR and poor outcome (mRS ≥ 3), and **(C)** NLR and the occurrence of sICH/HT. HT, hemorrhagic transformation; mRS, modified Rankin Scale; NLR, neutrophil-to-lymphocyte ratio; sICH, spontaneous intracerebral hemorrhage.

There were 19 studies with 11,124 patients that reported the association between acute ischemic stroke and mortality. After pooling the ORs, we found that the high NLR was correlated with an increased mortality of the AIS patients with an OR of 1.12 (95% CI, 1.07–1.16; *p* < 0.00001, Figure 2A) in a random-effect model, with evidence of moderate heterogeneity (τ^2^ <0.01; *I*^2^ = 76%; *p* < 0.00001).

**Figure 3:**
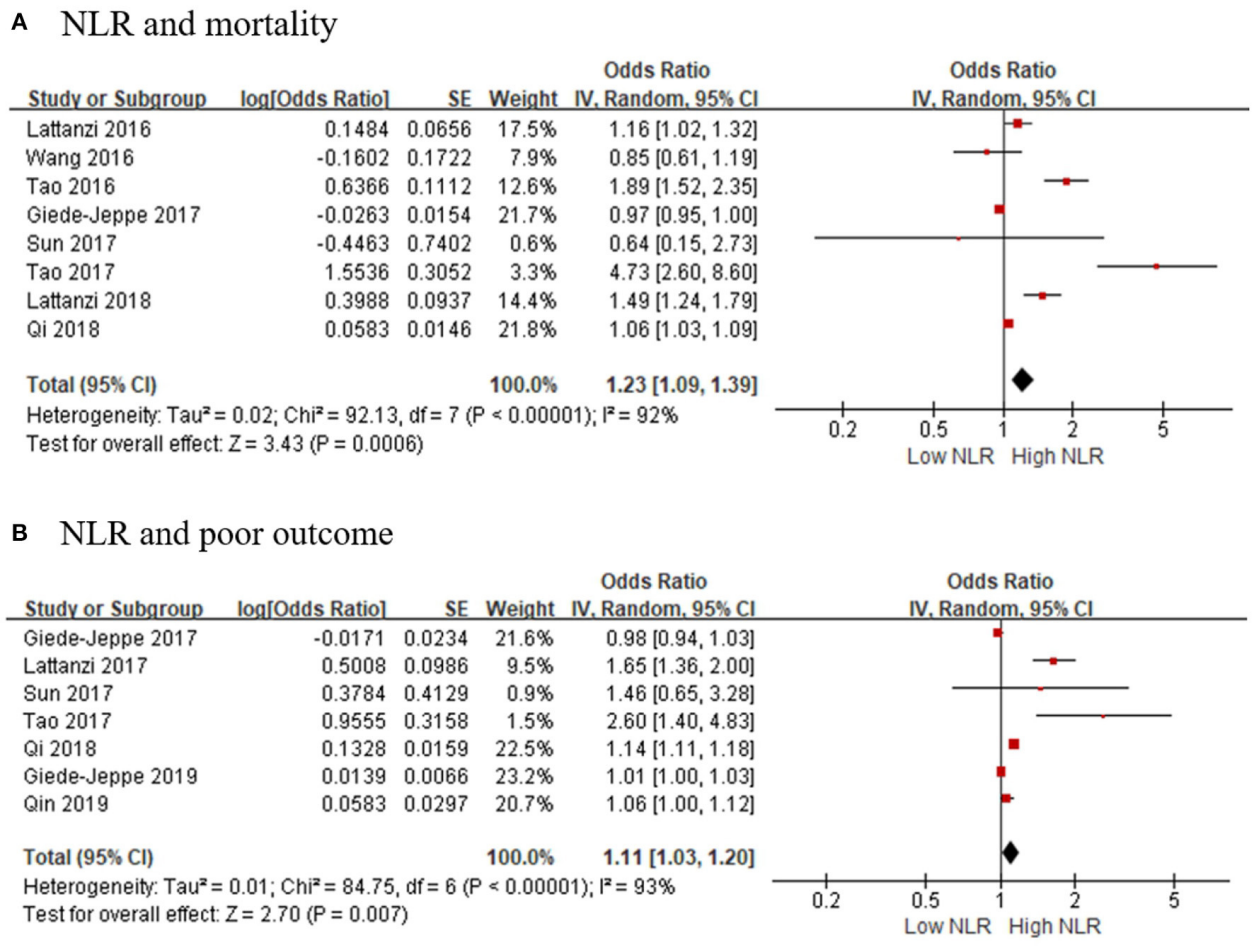
Forest plots of included studies evaluating the association in hemorrhagic stroke patients between **(A)** NLR and mortality, and **(B)** NLR and poor outcome (mRS ≥ 3). mRS, modified Rankin Scale; NLR, neutrophil-to-lymphocyte ratio.

As for hemorrhagic stroke, a total of eight articles with 2,957 participants were included in this meta-analysis. The result showed that the higher risk of death was associated with high NLR, and the pooled OR was 1.23 (95% CI, 1.09–1.39; *p* = 0.0006, Figure 3A), using a random-effect model. Significant heterogeneity between the eight studies was observed (τ^2^= 0.02; *I*^2^ = 92%; *p* < 0.00001).

#### Association of Neutrophil-to-Lymphocyte Ratio and Poor Outcomes

Fifteen studies showed the relationship of NLR and poor outcomes in 5,679 AIS patients. As shown in Figure 2B, the gathered OR was 1.29 (95% CI, 1.16–1.44; *p* < 0.00001) in a random-effect model, which means the higher the NLR is, the poorer the outcomes are. The heterogeneity detected between the articles was τ^2^= 0.03; *I*^2^ = 82%; *p* < 0.00001.

We selected seven articles to explore the connection of high NLR and poor outcomes in patients with hemorrhagic stroke. The pooled OR was 1.11 (95% CI, 1.03–1.20; *p* = 0.007, Figure 3B), suggesting that poor outcomes in patients with AHS is associated with a higher NLR. Heterogeneity among the studies was τ^2^= 0.01; *I*^2^ = 93%; *p* < 0.00001.

#### Association of Neutrophil-to-Lymphocyte Ratio and Hemorrhagic Transformation or Spontaneous Intracerebral Hemorrhage

Eleven articles with 4,539 patients provided ORs and 95% CI for the risk factor for HT or sICH. Figure 2C shows a significant correlation between NLR and HT or sICH rates in patients with AIS, with a pooled OR of 1.15 (95% CI, 1.08–1.23; *p* < 0.0001). The heterogeneity discovered between the articles was τ^2^= 0.01; *I*^2^ = 69%; *p* < 0.0001.

### Subgroup Prognostic Analyses

#### Subgroup Analysis of Mortality

Subgroup analysis of mortality in AIS patients is demonstrated in Table 3. We classified the mortality as follows: region, sample of sizes, treatment methods, cutoff value, median or mean age, stroke severity, follow-up period, data type, study type, and Newcastle–Ottawa Scale (NOS) quality scores. In general, elevated NLR value and higher risk of death in AIS patients were viewed constantly in all subgroups, except for in-hospital mortality. In the subgroup based on region, Asia and non-Asia groups were both observed to be associated with high NLR, with the pooled OR being 1.08 (95% CI, 1.03–1.12, *p* = 0.0006) and 1.19 (95% CI, 1.10–1.28, *p* < 0.0001), respectively. Cohorts with EVT were more likely to have a risk of death, with an OR of 1.29 (95% CI, 1.11–1.50, *p* = 0.0009). Furthermore, stroke severity, follow-up period, and study type could be the potential source of heterogeneity.

**Table 3:** Subgroup analysis for NLR in AIS patients.

**Stratified analyses**	**No. of studies**	**No. of patients**	**Pooled ORs (95%CI)**	***p*-Value**	**Heterogeneity**	
					***I*^**2**^ (%)**	***p*_**H**_-Value**
**Mortality**						
Overall analysis	19	11,124	1.12 (1.07–1.16)	<0.00001	76	<0.00001
*Region*						
Asian	8	4,047	1.08 (1.03–1.12)	0.0006	74	0.0002
Non-Asian	11	7,077	1.19 (1.10–1.28)	<0.0001	72	<0.0001
*Study size*						
≥500	7	5,168	1.10 (1.05–1.15)	<0.0001	65	0.008
<500	12	5,956	1.16 (1.08–1.24)	<0.0001	79	<0.00001
*Therapy*						
EVT	7	3,333	1.29 (1.11–1.50)	0.0009	80	<0.0001
Non-EVT	10	4,280	1.13 (1.05–1.21)	0.0007	68	0.0006
*Age*						
≥65	11	6,637	1.19 (1.11–1.28)	<0.00001	71	<0.0001
<65	8	2,756	1.07 (1.01–1.13)	0.01	67	0.002
*Baseline NIHSS score*						
NIHSS ≥ 10	7	2,568	1.09 (1.06–1.13)	<0.00001	42	0.11
NIHSS <10	5	4,975	1.06 (1.04–1.07)	<0.00001	86	<0.00001
*Assessment time*						
In-hospital	3	2,251	1.06 (0.99–1.14)	0.08	80	0.007
≤ 1 month	2	287	1.16 (1.08–1.25)	<0.0001	37	0.21
1–3 months	14	8,586	1.16 (1.09–1.24)	<0.00001	67	<0.0001
1 year	1	–	–	–	–	–
*Variable type*						
Categorical	6	2,944	1.84 (1.13–3.00)	0.01	73	0.002
Continuous	13	8,180	1.11 (1.07–1.16)	<0.00001	78	<0.00001
*Study type*						
Prospective	3	1,354	1.08 (1.04–1.13)	0.0003	22	0.28
Retrospective	16	9,770	1.13 (1.08–1.18)	<0.00001	78	<0.00001
*Cutoff value*						
≥7	10	5,168	1.13 (1.10–1.17)	<0.00001	39	0.10
<7	9	5,956	1.08 (1.03–1.13)	0.0009	77	<0.00001
*NOS score*						
≥7	16	8,854	1.12 (1.07–1.17)	<0.00001	78	<0.00001
<7	3	2,270	1.10 (1.04–1.17)	0.002	25	0.27
**Poor outcome (mRS** **≥** **3)**						
Overall Analysis	15	5,679	1.29 (1.16–1.44)	<0.00001	82	<0.00001
*Region*						
Asian	9	3,508	1.37 (1.17–1.61)	<0.0001	82	<0.00001
Non-Asian	6	2,171	1.37 (1.11–1.68)	0.003	87	<0.00001
*Study size*						
≥500	5	3,224	1.24 (1.01–1.52)	0.04	87	<0.00001
<500	10	2,455	1.36 (1.18–1.58)	<0.0001	77	<0.00001
*Therapy*						
EVT	8	3,027	1.32 (1.14–1.51)	<0.0001	77	<0.00001
Non-EVT	6	2,548	1.30 (1.05–1.62)	0.02	87	<0.00001
*Age*						
≥65	9	3,445	1.46 (1.20–1.77)	<0.00001	85	0.0002
<65	5	2,130	1.11 (0.99–1.25)	0.07	76	0.002
*Baseline NIHSS score*						
NIHSS≥10	6	1,790	1.23 (1.07–1.41)	0.004	77	0.0006
NIHSS <10	5	2,397	1.61 (0.97–2.68)	0.07	90	<0.00001
*Variable type*						
Categorical	7	2,723	1.94 (1.59–2.36)	<0.00001	15	0.32
Continuous	8	2,956	1.13 (1.03–1.25)	0.01	80	<0.00001
*Study type*						
Prospective	3	684	1.48 (0.92–2.38)	0.11	73	0.005
Retrospective	10	4,560	1.19 (1.07–1.32)	0.002	83	<0.00001
*Cutoff value*						
≥7	9	3,263	1.22 (1.06–1.40)	0.005	72	0.0004
<7	6	2,416	1.73 (1.49–2.01)	<0.00001	26	0.23
*NOS score*						
≥7	13	4,255	1.36 (1.19–1.56)	<0.00001	82	<0.00001
<7	2	1,424	1.18 (0.75–1.87)	0.47	78	0.03
**sICH/HT**						
Overall analysis	11	4,539	1.15 (1.08–1.23)	<0.0001	69	<0.0001
*Region*						
Asian	5	2,219	1.13 (1.02–1.25)	0.02	67	0.01
Non-Asian	6	2,320	1.18 (1.07–1.30)	0.001	69	0.003
*Study size*						
≥500	4	2,780	1.32 (1.05–1.66)	0.02	89	<0.00001
<500	7	1,759	1.12 (1.08–1.17)	<0.00001	6	0.39
*Therapy*						
EVT	8	2,823	1.20 (1.09–1.32)	0.0002	65	0.003
Non-EVT	2	1,318	1.10 (0.94–1.28)	0.23	88	0.003
*Age*						
≥65	7	2,851	1.27 (1.11–1.44)	0.0004	68	0.001
<65	2	1,101	1.05 (0.98–1.15)	0.17	70	0.07
*Baseline NIHSS score*						
NIHSS≥10	6	2,426	1.22 (1.07–1.38)	0.003	72	0.001
NIHSS <10	1	510	–	–	–	–
*Variable type*						
Categorical	2	1,462	2.55 (1.29–1.60)	0.007	64	0.09
Continuous	9	3,077	1.12 (1.06–1.18)	<0.0001	53	0.02
*Study type*						
Prospective	4	1,641	1.31 (1.07–1.60)	0.009	76	0.002
Retrospective	7	2,898	1.12 (1.05–1.20)	0.0009	64	0.007
*Cut-off value*						
≥7	5	2,159	1.18 (1.10–1.27)	<0.0001	48	0.10
<7	6	2,380	1.06 (1.03–1.10)	0.0004	31	0.20
*NOS score*						
≥7	8	2,269	1.13 (1.09–1.18)	<0.00001	8	0.36
<7	3	2,270	1.82 (0.85–3.89)	0.12	90	<0.0001

“*-”, No such data*.

*AIS, acute ischemic stroke; HT, hemorrhagic transformation; mRS, modified Rankin Scale; NLR, neutrophil-to-lymphocyte ratio; sICH, spontaneous intracerebral hemorrhage; OR, odds ratio; CI, confidence interval; EVT, endovascular therapy; NIHSS, National Institutes of Health Stroke Scale; NOS, Newcastle–Ottawa Scale*.

We further carried out another subgroup of mortality in AHS patients. The subgroup was stratified by the before-mentioned criterion. As shown in Table 4, we found that the region of study and age of patients with elevated NLR were not related to mortality in AHS patients. Generally speaking, stroke severity could be a potential source of heterogeneity.

**Table 4:** Subgroup analysis for NLR in AHS patients.

**Stratified analyses**	**No. of studies**	**No. of patients**	**Pooled ORs (95% CI)**	***p*-Value**	**Heterogeneity**	
					***I^**2**^* (%)**	***p*_**H**_-value**
**Mortality**						
Overall Analysis	8	2,957	1.23 (1.09–1.39)	0.0006	92	<0.00001
*Region*						
Asian	5	1,717	1.47 (0.94–2.30)	0.09	92	<0.00001
Non-Asian	3	1,240	1.17 (0.93–1.47)	0.17	92	<0.00001
*Study size*						
≥500	2	1,413	1.02 (0.94–1.10)	0.70	94	<0.0001
<500	6	1,544	1.50 (1.09–2.06)	0.01	88	<0.00001
*Age*						
≥65	3	609	1.18 (0.91–1.51)	0.21	79	0.009
<65	4	1,493	1.74 (0.98–3.09)	0.06	94	<0.00001
*Baseline NIHSS score*						
NIHSS≥10	2	5,60	1.97 (0.37–10.56)	0.43	96	<0.00001
NIHSS <10	3	1,087	1.06 (1.04–1.09)	<0.0001	12	0.32
*Assessment time*						
≤ 1 month	2	432	1.15 (0.66–1.98)	0.62	88	0.004
1–3 months	6	2,525	1.22 (1.08–1.38)	0.002	93	<0.00001
*Variable type*						
Categorical	2	688	1.95 (0.28–13.66)	0.50	84	0.01
Continuous	6	2,269	1.17 (1.05–1.30)	0.004	93	<0.00001
*Study type*						
Prospective	1	247	–	–	–	–
Retrospective	6	2,486	1.07 (1.04–1.31)	0.007	92	<0.00001
*Cutoff value*						
≥7	5	1,897	1.20 (1.04–1.38)	0.01	95	<0.00001
<7	3	1,060	1.28 (1.01–1.62)	0.04	64	0.02
*NOS score*						
≥7	7	2,399	1.37 (1.06–1.78)	0.02	93	<0.00001
<7	1	558	–	–	–	–
**Poor outcome (mRS** **≥** **3)**						
Overall Analysis	7	2,825	1.11 (1.03–1.20)	0.007	93	<0.00001
*Region*						
Asian	4	1,459	1.13 (1.02–1.26)	0.02	76	0.006
Non-Asian	3	1,366	1.01 (1.00–1.02)	0.07	38	0.20
*Age*						
≥65	2	1,413	1.06 (0.92–1.23)	0.43	96	<0.00001
<65	5	1,412	1.20 (1.05–1.38)	0.009	89	<0.00001
*Baseline NIHSS score*						
NIHSS ≥ 10	1	558	–	–	–	–
NIHSS <10	4	1,459	1.13 (1.02–1.26)	0.02	76	0.006
*Age*						
≥65	2	565	1.06 (1.00–1.13)	0.04	0	0.44
<65	2	528	1.72 (1.43–2.07)	<0.00001	47	0.17
*Variable type*						
Categorical	5	1,778	1.09 (1.00–1.20)	0.05	93	<0.00001
Continuous	2	1,047	1.26 (0.76–2.10)	0.37	96	<0.00001
*Study type*						
Prospective	1	319	–	–	–	–
Retrospective	6	2,506	1.17 (1.05–1.31)	0.006	91	<0.00001
*Cutoff value*						
≥7	2	865	1.14 (1.11–1.18)	<0.00001	0	0.55
<7	3	1,025	1.72 (1.43–2.07)	<0.00001	47	0.17
*NOS score*						
≥7	6	2,267	1.10 (1.01–1.19)	0.03	87	<0.00001
<7	1	558	-	-	-	-

“*–”, No such data*.

*AHS, acute hemorrhagic stroke; mRS, modified Rankin Scale; NLR, neutrophil-to-lymphocyte ratio; NIHSS, National Institutes of Health Stroke Scale; NOS, Newcastle–Ottawa Scale*.

#### Subgroup Analysis of Poor Outcomes

Subgroup analysis of poor outcomes in AIS patients revealed that elevated NLR was significantly associated with poor outcomes in studies performed by categorical variables. In addition, when stratified by the study data type, heterogeneity was evidently reduced in categorical variables, meaning the study data type may be the resource of heterogeneity (Table 3).

Table 4 shows the subgroup of the relationship between NLR and poor outcomes in AHS patients. Interestingly, we found that poor outcomes could be significantly associated with stroke severity in both the NIHSS score ≥ 10 and NIHSS score <10 subgroups, because the heterogeneity in the subgroups was both shrunk.

#### Subgroup Analysis of Hemorrhagic Transformation or Spontaneous Intracerebral Hemorrhage

Subgroup based on age revealed that the NLR had considerable effect on occurrence of HT in elderly individuals with an OR of 1.27 (95% CI, 1.11–1.44, *p* = 0.0004), whereas no significant association was observed in studies in non-elderly individuals. Interestingly, heterogeneity was obviously decreased after stratifying the cutoff value of NLR, and the results showed that NLR was closely related to HT in both the cutoff value of NLR > 7 and cutoff value ≤ 7 subgroups. In addition, in the small sample size (*n* < 500) subgroup, the heterogeneity was evidently reduced (*I*^2^ = 6, *p* = 0.39), meaning the sample size could be the hidden origin of heterogeneity (Table 3).

### Publication Bias

**Figure 4:**
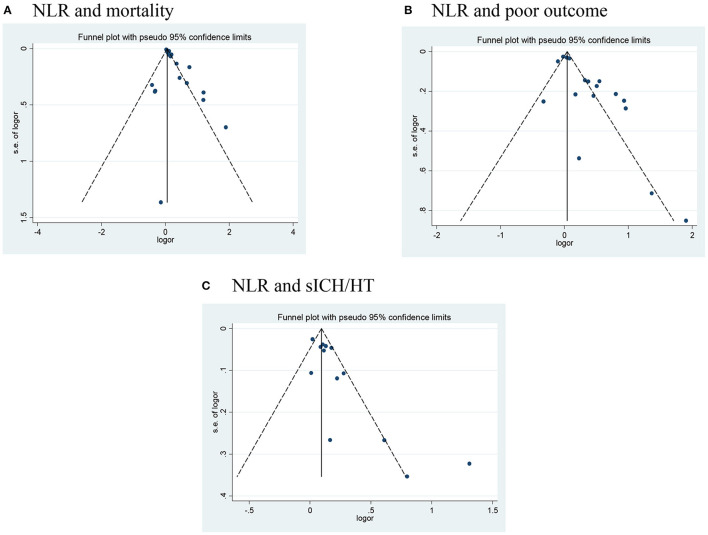
Funnel plots of **(A)** NLR and mortality, **(B)** NLR and poor outcome (mRS ≥ 3), and **(C)** NLR and the occurrence of sICH/HT in ischemic stroke. HT, hemorrhagic transformation; mRS, modified Rankin Scale; NLR, neutrophil-to-lymphocyte ratio; sICH, spontaneous intracerebral hemorrhage.

**Figure 5:**
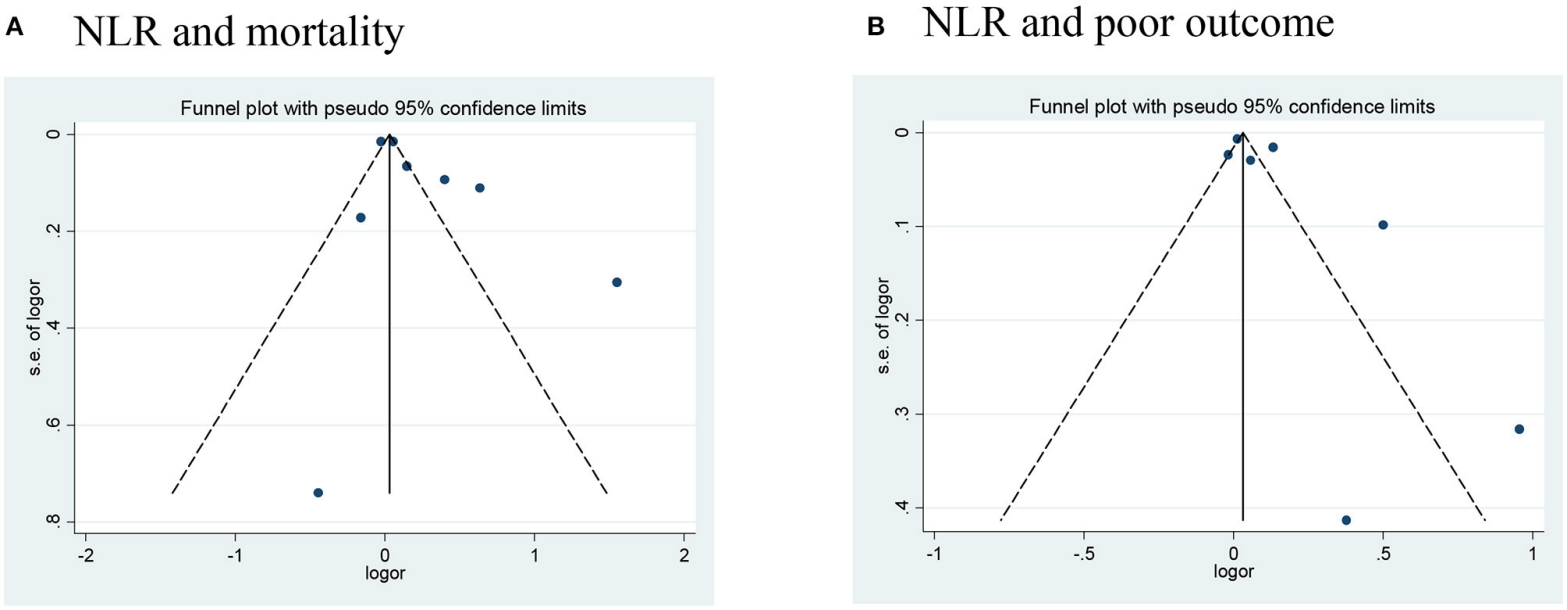
Funnel plots of **(A)** NLR and mortality, and **(B)** NLR and poor outcome (mRS ≥ 3) in hemorrhagic stroke. mRS, modified Rankin Scale; NLR, neutrophil-to-lymphocyte ratio.

Publication bias was assessed in studies that provided outcomes in both AIS and AHS patients. After performing funnel plots, we found significant bias in ischemic stroke because the funnel plots were asymmetric (Figure 4). Furthermore, Egger's tests indicated some degree of publication bias (both *p* < 0.05, Supplementary Figure 1). Then, the trim and fill method was applied to solve these problems. After the adjustment, the results showed that high NLR was associated with mortality, poor outcome (mRS ≥ 3), and the occurrence of sICH or HT with adjusted ORs of 1.14 (95% CI, 1.07–1.16, *p* < 0.0001), 1.10 (95% CI, 0.99–1.23, *p* = 0.088), and 1.10 (95% CI, 1.02–1.87, *p* = 0.012), respectively. As for bias in hemorrhagic stroke, although the funnel plots were not completely symmetrical (Figure 5), the Egger's test does not suggest significant bias (Supplementary Figure 2). Sensitivity analysis was performed by excluding the included studies one by one (Supplementary Tables 2, 3), and the results did not change significantly from before deletion, suggesting that the results of this study were relatively stable.

## Discussion

Stroke is one of the important diseases that seriously threaten human health, which has high mortality and morbidity (50). Despite its high incidence, there are few effective treatments to improve the quality of life of patients. Therefore, it is of great clinical significance to find simple and accurate serum biomarkers to assess the degree of early neurological damage and prognosis of stroke patients. The purpose of this meta-analysis was to evaluate the prognostic of NLR in patients with stroke. Inflammation is considered to be the secondary damage mechanism of stroke. We carried out the meta-analysis including 41 articles based on criterion with 27,124 individuals, and the result revealed that NLR was independently associated with prognosis in patients with stroke.

In recent years, inflammation has shown to have a strong relationship with the occurrence of stroke (51). Post-stroke inflammation has a harmful effect on brain injury, but it may play a protective role in tissue restoration and regeneration, and its role changes over time (52). As the marker of systemic inflammation, white blood cell counts were significantly increased after stroke (53) and related to the poor prognosis of stroke patients (54, 55). As we all know, different subtypes of WBC may have different effects on the inflammatory response of damaged tissues. Neutrophils are the major subtype of white blood cells that can respond earlier after stroke and show an active inflammatory response (52). Neutrophils first accumulate in the cerebral blood vessels within a few hours, which may cause the expansion of the infarction and block the microvessels (56). On the other hand, neutrophils increase the expression of matrix metalloprotein 9, which directly destroys the blood–brain barrier, causing secondary brain injury or hemorrhagic transformation (57, 58). Previous studies have confirmed that early leukocytosis and neutrophilia are related to the infarct volume assessed by DWI in the early stage of ischemic stroke (59). Neutrophils are recruited to the ischemic area of brain tissue and may release some proteolytic enzymes or free oxygen free radicals and other inflammatory mediators into the damaged area (5). It has recently been reported that when patients with acute cerebral infarction are admitted to the hospital, a higher total number of white blood cells and neutrophil counts are associated with the severity of stroke (60). Similar with cerebral infarction, some studies showed that elevated neutrophil level was related to hematoma volume and outcomes in ICH patients. Moreover, studies revealed that in a rat autologous blood model, neutrophils penetrated in and around the hematoma, which reached a peak at 2–3 days (61). Activated neutrophils can release a variety of proteolytic enzymes and pro-inflammatory proteases, which can damage the brain tissue directly.

Subpopulations of lymphocytes, especially T lymphocytes, may have regulatory functions in inflammation-induced neuroprotection (62). Lymphocytes in ischemic brain tissue rise later than neutrophils. Lymphocytes have been found to play an important role in healing or repairing inflammation (63). However, the role of lymphocytes in the pathogenesis of stroke remains controversial. It has been reported that other subtypes of T cells (pro-inflammatory lymphocytes) are the main source of cytotoxic substances and have a negative effect on ischemic brain tissue (64). Then, there is also experimental evidence that certain subtypes of lymphocytes (mainly regulatory T cells and B cells) have regulatory functions, and these cells are responsible for the reduction of ischemic tissue volume in ischemic stroke and the improvement of function after neurological deficit (65). Early studies indicated that a higher level of lymphocytes could upregulate the anti-inflammatory cytokine interleukin (IL)-10 and suppress inflammatory cytokines including tumor necrosis factor (TNF)-α and IL-6, which can play an anti-inflammatory effect (66). In addition, clinical evidence shows that lower lymphocyte count is associated with poor early neurological function improvement and poor long-term functional prognosis (67).

NLR stands for an easily available, non-invasive, and inexpensive marker that can be routinely used to evaluate systemic inflammation in clinical work. The mechanism behind the clinical significance of NLR in stroke is that inflammation plays a central role in all types of stroke, from the occurrence and development of injury to recovery (68). The underlying mechanism of elevated NLR and poor prognosis may be related to excessive activation of inflammation and immunosuppression (69). First, after an ischemic stroke, the damaged brain tissue will produce a strong inflammatory response and, consequently, produce inflammatory biomarkers (69). Although inflammation is necessary for early repair after stroke, excessive activation of inflammation can cause damage to the brain tissue, leading to deterioration of neurological function and brain edema (70, 71). Second, the theories about immunosuppression suggest that after a stroke, catecholamines are released into the blood through over-activated sympathetic nerves, which may reduce circulating lymphocyte level and increase the risk of infection (71). Recently, NLR has been proposed as an independent predictor of severity and mortality to predict coronary syndrome (72).

Application prognostic biomarkers may enhance risk stratification, help design individual treatment, and determine follow-up schedules. In the stage of customized treatment strategy, NLR may be a key sticking point in the risk stratification of acute stroke patients. Moreover, neurologists can develop more frequent and stricter follow-up strategies for patients who may have a poor prognosis. In short, in the different stages of diagnosis, treatment, and follow-up, the application of these preoperative lymphocyte-related systemic inflammation biomarkers may improve the accuracy of current prognostic models and help make clinical decisions.

This meta-analysis adopted strict inclusion and exclusion criteria, and covered 41 medium-to-high-quality studies, including retrospective studies and prospective studies, and successively explored the relationship between NLR and the prognosis of ischemic stroke and hemorrhagic stroke. Our meta-analysis found that high NLR has a predictive effect on the prognosis in stroke patients, mainly in terms of mortality, poor prognosis, and hemorrhagic transformation. Meanwhile, NLR has a stronger predictive effect on ischemic stroke than hemorrhagic stroke. Many studies also support that NLR is an independent risk factor for predicting short-term outcomes in both ischemic and hemorrhagic stroke. Zhang et al. (11) reported that NLR was the best independent predictor associated with mortality and poor outcome in AIS patients. Ying et al. (14) suggested that NLR could predict the HT and outcome in AIS patients with r-tPA treatment. Switonska et al. (15) indicated that NLR is an inexpensive tool that could identify the increased risk of early symptomatic hemorrhage after recanalization in AIS. Giede-Jeppe et al. (38) proved that in aSAH patients, NLR represents an independent parameter associated with unfavorable functional outcome. For NLR and AIS, the results of meta-analysis show that NLR has a significant correlation with the two. In the AHS patients, although the meta-analysis results suggest that patients with higher NLR have a poorer prognosis, the heterogeneity between the studies is high, and no single article that can significantly reduce the heterogeneity was found in the sensitivity analysis. In the subsequent subgroup analysis, the source of heterogeneity was deeply explored, and the stability of the results was further proved. Furthermore, we found that a high NLR is more closely related to the prognosis of AIS patients after endovascular treatment. Therefore, as a potential prognostic biomarker, NLR will help to more accurately determine the prognosis of stroke patients.

There are also some limitations in this study: First, the inflammatory process is relatively complex. We use a relatively simple ratio of neutrophils to lymphocytes to show the effect of inflammation on the prognosis of patients with stroke. It only reflects the general trend, not the full picture of the inflammatory process. Second, the results of some subgroup analysis suggest that there is a high degree of heterogeneity among the studies, and there may be potential selection bias or other confounding factors. Third, the ORs obtained by this meta-analysis are small; this requires clinicians to use it with caution and explain carefully. It is necessary to combine the patient's own situation to predict the prognosis of acute stroke patients. Moreover, there were few studies with negative results in this meta-analysis, which might lead to potential publication bias. Besides, only two subarachnoid hemorrhage cases were included in this meta-analysis that might result in an inaccuracy of the results. We look forward to more researches to deeply discuss this relationship. Finally, the optimal cutoff values of NLR remain undetermined. In this study, the NLR cutoff values of each article are different, and different NLR cutoff values may cause higher heterogeneity, which may interfere with the accuracy of our analysis results. Therefore, the establishment of a standard NLR cutoff value will promote in-depth research on its prognostic value.

## Conclusion

In summary, our research refers that a high NLR value is closely related to the prognosis of stroke patients. High NLR is associated with a 1.1- to 1.3-fold increased risk of poor outcomes of AIS/AHS patients. Elevated NLR can predict the mortality, poor prognosis, and the occurrence of spontaneous cerebral hemorrhage in stroke patients, and our subgroup analysis suggests that a high NLR is more closely related to the prognosis of AIS patients after endovascular treatment. This low-cost and easy-to-obtain biomarker will play a greater and more profound role in clinical work in the future. Future studies need to combine with TOAST classification, OCSB classification, and other indicators in order to better predict the prognosis of stroke patients.

## Data Availability Statement

The raw data supporting the conclusions of this article will be made available by the authors, without undue reservation.

## Author Contributions

WL and MH conceived the presented idea and designed the research. ZD and XL searched databases and performed data analysis and statistical analysis. WL, MH, YS, and XL were responsible for writing the manuscript. All authors critically revised the article for important intellectual content and approved the final manuscript.

## Funding

This work was supported by the Key Research and Development Program, Science and Technology Department of Shanxi Province (grant number 201703D421018), Selected Program for Science and Technology Activities of Overseas Students, Science and Technology Department of Shanxi Province (grant number 2018-1059-13), Scientific Research Program for Returned Overseas Students, Science and Technology Department of Shanxi Province (grant number HGKY2019096), and Talent introduces research launches of Shanxi Bethune Hospital (grant number 2020RC007).

## Conflict of Interest

The authors declare that the research was conducted in the absence of any commercial or financial relationships that could be construed as a potential conflict of interest.

## Publisher's Note

All claims expressed in this article are solely those of the authors and do not necessarily represent those of their affiliated organizations, or those of the publisher, the editors and the reviewers. Any product that may be evaluated in this article, or claim that may be made by its manufacturer, is not guaranteed or endorsed by the publisher.

## Supplementary Material

Supplementary Figure 1 Publication bias assessment with Egger's tests for the outcomes in ischemic stroke, **(a)** NLR and mortality, **(b)** NLR and poor outcome (mRS ≥ 3), and **(c)** NLR and the occurrence of sICH/HT in ischemic stroke. HT, hemorrhagic transformation, mRS, modified Rankin Scale, NLR, neutrophil-to-lymphocyte ratio, sICH, spontaneous intracerebral hemorrhage.

Supplementary Figure 2 Publication bias assessment with Egger's tests for the outcomes in hemorrhagic stroke, **(a)** NLR and mortality, **(b)** NLR and poor outcome (mRS ≥ 3). mRS, modified Rankin Scale, NLR, neutrophil-to-lymphocyte ratio.

Supplementary Table 1 Search strategy of present systematic review and meta-analysis.

Supplementary Table 2 Sensitivity analyses for NLR in AIS patients. AIS, acute ischemic stroke, NLR, neutrophil-to-lymphocyte ratio, sICH, spontaneous intracerebral hemorrhage, OA, On admission, OR, Odds ratio, CI, confidence interval, DFS, disease-free survival, OS, overall survival.

Supplementary Table 3 Sensitivity analyses for NLR in AHS patients. AHS, acute hemorrhagic stroke, mRS, modifie.

## Abbreviations

AIS: acute ischemic stroke
AHS: acute hemorrhagic stroke
CE: cardio embolism
CI: confidence interval
CAD: coronary artery disease
DM: diabetes mellitus
DFS: disease-free survival
EVT: endovascular therapy
GCS: Glasgow Coma Score
HT: hemorrhagic transformation
HBP: high blood pressure
IVT: intravenous thrombolysis
IQR: interquartile range
LAA: large artery atherosclerosis
LACI: lacunar cerebral infarction
LHI: large hemispheric infarction
mRS: modified Rankin Scale
MT: mechanical thrombectomy
NLR: neutrophil-to-lymphocyte ratio
NIHSS: National Institutes of Health Stroke Scale
NA: not available
Non: none
NOS: Newcastle-Ottawa Scale
OR: Odds ratio
OA: On admission
OS: overall survival
PACI: partial anterior cerebral infarction
POCI: posterior cerebral infarction
RCIHS: recurrent cerebral ischemic hemispheric stroke
SVO: small vessel occlusion
SUC: stroke of undetermined cause
SOE: stroke of other determined etiology
sICH: spontaneous intracerebral hemorrhage
SAH: subarachnoid hemorrhage
SSS: Scandinavian Stroke Scale
TACI: total anterior cerebral infarction.
