# Supplementary figures and images for: Prognostic Value of Neutrophil-to-Lymphocyte Ratio in Stroke: A Systematic Review and Meta-Analysis
Source: Front Neurol. 2021 Sep 24;12:686983. doi: 10.3389/fneur.2021.686983 (PMC8497704; doi:10.3389/fneur.2021.686983)

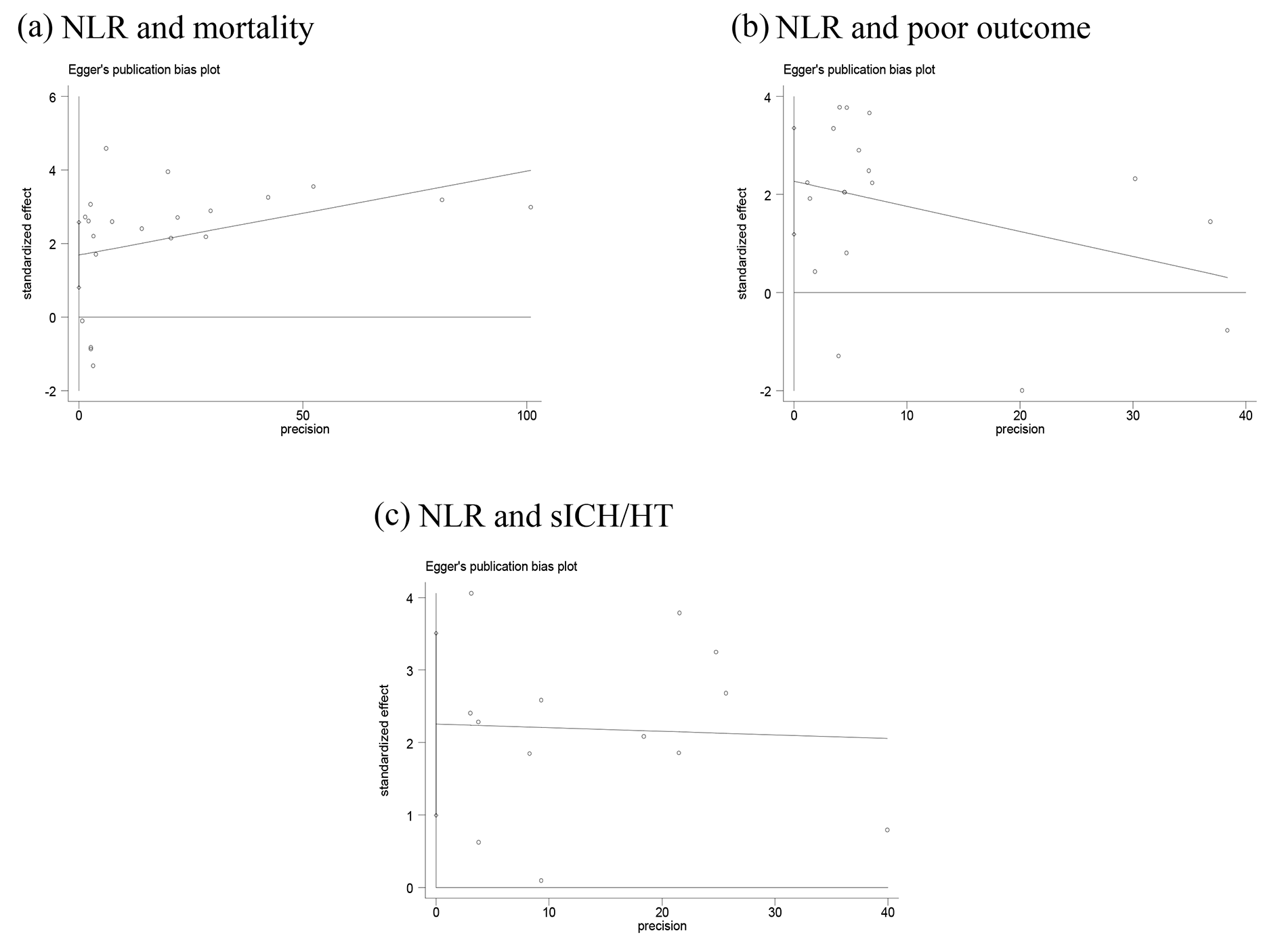

Supplement: Supplementary Figure 1 — Publication bias assessment with Egger's tests for the outcomes in ischemic stroke, (a) NLR and mortality, (b) NLR and poor outcome (mRS ≥ 3), and (c) NLR and the occurrence of sICH/HT in ischemic stroke. HT, hemorrhagic transformation, mRS, modified Rankin Scale, NLR, neutrophil-to-lymphocyte ratio, sICH, spontaneous intracerebral hemorrhage. [file Image_1.TIF]

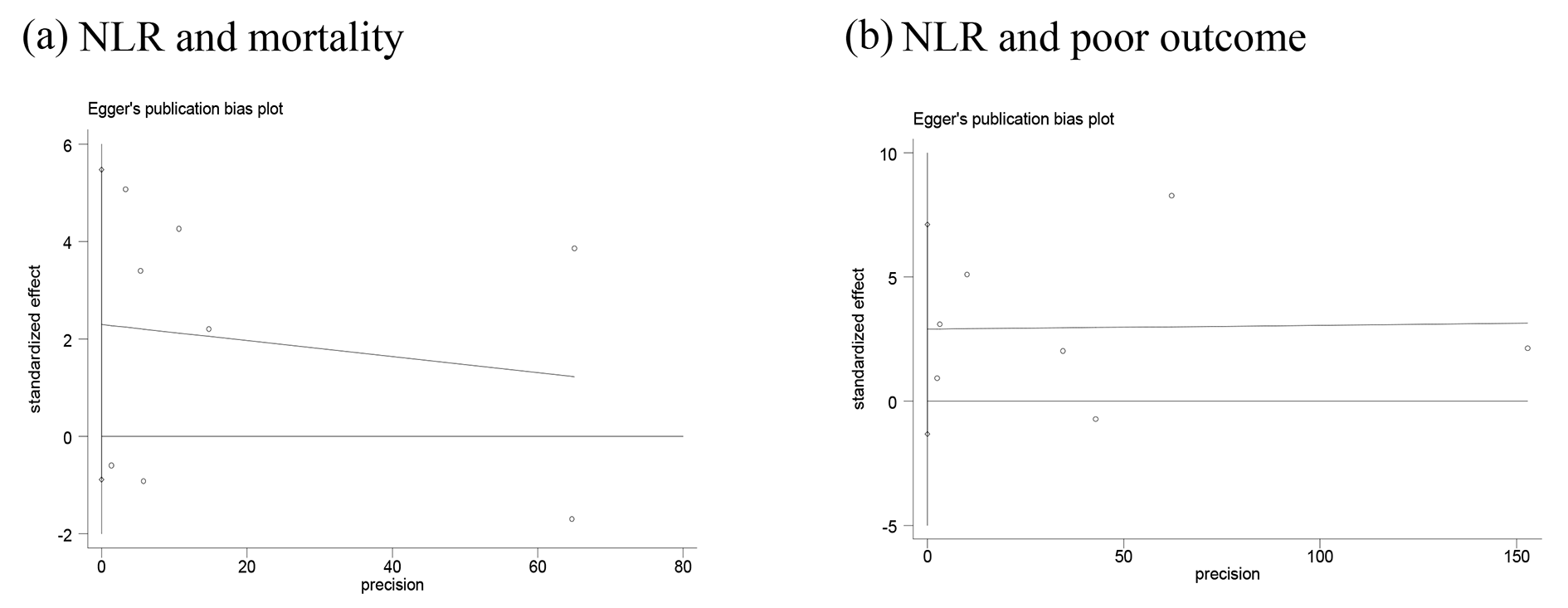

Supplement: Supplementary Figure 2 — Publication bias assessment with Egger's tests for the outcomes in hemorrhagic stroke, (a) NLR and mortality, (b) NLR and poor outcome (mRS ≥ 3). mRS, modified Rankin Scale, NLR, neutrophil-to-lymphocyte ratio. [file Image_2.TIF]
