# Supplementary material for: Prognostic Value of Neutrophil-to-Lymphocyte Ratio in Stroke: A Systematic Review and Meta-Analysis
Source: Front Neurol. 2021 Sep 24;12:686983. doi: 10.3389/fneur.2021.686983 (PMC8497704; doi:10.3389/fneur.2021.686983)
Supplement: Supplementary Table 1 — Search strategy of present systematic review and meta-analysis. [file Table_1.DOCX]

| **Search strategy** | |
| --- | --- |
| PubMed | ((NLR) OR (neutrophil-to-lymphocyte ratio) OR (neutrophil-lymphocyte ratio)) AND ((stroke) OR (acute ischemic stroke) OR (cerebrovascular accident) OR (CVA) OR (AIS) OR (TIA) OR (intra-cerebral hemorrhage) OR (intracranial hemorrhage) OR (AHS) OR (subarachnoid hemorrhage)) |
| Web of science | ALL=(“NLR” OR “neutrophil-to-lymphocyte ratio” OR “neutrophil-lymphocyte ratio”) AND ALL=(“stroke” OR “acute ischemic stroke” OR “cerebrovascular accident” OR “CVA” OR “AIS” OR “TIA” OR “intra-cerebral hemorrhage” OR “intracranial hemorrhage” OR “AHS” OR “subarachnoid hemorrhage”) |
| Embase (Ovid SP): | (“NLR” OR “neutrophil-to-lymphocyte ratio” OR “neutrophil-lymphocyte ratio”) AND (“stroke” OR “acute ischemic stroke” OR “cerebrovascular accident” OR “CVA” OR “AIS” OR “TIA” OR “intra-cerebral hemorrhage” OR “intracranial hemorrhage” OR “AHS” OR “subarachnoid hemorrhage”) |
| Chochrane: | (ALL (“NLR”) OR ALL (“neutrophil-to-lymphocyte ratio”) OR ALL(“neutrophil-lymphocyte ratio”)) AND (ALL (“stroke”) OR ALL (“acute ischemic stroke”) OR ALL (“cerebrovascular accident”) OR ALL (“CVA”) OR ALL (“AIS”) OR ALL (“TIA”) OR ALL (“intra-cerebral hemorrhage”) OR ALL (“intracranial hemorrhage”) OR ALL (“AHS”) OR ALL (“subarachnoid hemorrhage”)) |
| Google Scholar: | (“NLR” OR “neutrophil-to-lymphocyte ratio” OR “neutrophil-lymphocyte ratio”) AND (“stroke” OR “acute ischemic stroke” OR “cerebrovascular accident” OR “CVA” OR “AIS” OR “TIA” OR “intra-cerebral hemorrhage” OR “intracranial hemorrhage” OR “AHS” OR “subarachnoid hemorrhage”) |
