# Supplementary material for: Prognostic Value of Neutrophil-to-Lymphocyte Ratio in Stroke: A Systematic Review and Meta-Analysis
Source: Front Neurol. 2021 Sep 24;12:686983. doi: 10.3389/fneur.2021.686983 (PMC8497704; doi:10.3389/fneur.2021.686983)
Supplement: Supplementary Table 2 — Sensitivity analyses for NLR in AIS patients. AIS, acute ischemic stroke, NLR, neutrophil-to-lymphocyte ratio, sICH, spontaneous intracerebral hemorrhage, OA, On admission, OR, Odds ratio, CI, confidence interval, DFS, disease-free survival, OS, overall survival. [file Table_2.DOCX]

| **Sensitivity analysis for NLR in AIS patients.** | | | | | |
| --- | --- | --- | --- | --- | --- |
| Study omitted | OR (95% CI) | *P* value | Heterogeneity | | Effect model |
|  |  |  | I^2^ (%) | *P*_H_ value |  |
| **NLR and** **mortality** |  |  |  |  |  |
| Zhang 2020 [11] | 1.12 (1.07-1.17) | <0.00001 | 75 | <0.00001 | Random |
| Semerano 2020 [12] | 1.12 (1.07-1.17) | <0.00001 | 77 | <0.00001 | Random |
| Semerano 2019 (24H) [19] | 1.10 (1.07-1.14) | <0.00001 | 71 | <0.00001 | Random |
| Kozyolkin 2019 [21] | 1.12 (1.07-1.17) | <0.00001 | 77 | <0.00001 | Random |
| Wang 2019 [17] | 1.12 (1.07-1.17) | <0.00001 | 76 | <0.00001 | Random |
| Sun 2019 [18] | 1.11 (1.07-1.16) | <0.00001 | 74 | <0.00001 | Random |
| Kocaturk 2019 [22] | 1.11 (1.07-1.16) | <0.00001 | 76 | <0.00001 | Random |
| Shi 2018 [10] | 1.12 (1.08-1.17) | <0.00001 | 76 | <0.00001 | Random |
| Duan 2018 [24] | 1.12 (1.07-1.16) | <0.00001 | 76 | <0.00001 | Random |
| Goyal 2018 [25] | 1.12 (1.08-1.17) | <0.00001 | 76 | <0.00001 | Random |
| Malhotra 2018 [26] | 1.11 (1.07-1.16) | <0.00001 | 76 | <0.00001 | Random |
| Yu 2018 [29] | 1.12 (1.07-1.16) | <0.00001 | 77 | <0.00001 | Random |
| Yilmaz 2017 (DFS) [9] | 1.12 (1.08-1.16) | <0.00001 | 77 | <0.00001 | Random |
| Yilmaz 2017 (OS) [9] | 1.12 (1.08-1.17) | <0.00001 | 77 | <0.00001 | Random |
| Fan 2017 (In-hospital) [32] | 1.13 (1.08-1.18) | <0.00001 | 77 | <0.00001 | Random |
| Fan 2017 (One-year) [32] | 1.13 (1.08-1.19) | <0.00001 | 72 | <0.00001 | Random |
| Fang 2017 [33] | 1.13 (1.08-1.19) | <0.00001 | 76 | <0.00001 | Random |
| Maestrini 2015 [35] | 1.13 (1.08-1.18) | <0.00001 | 77 | <0.00001 | Random |
| Brooks 2014 [36] | 1.11 (1.07-1.16) | <0.00001 | 75 | <0.00001 | Random |
| Tokgoz 2014 [37] | 1.11 (1.07-1.16) | <0.00001 | 75 | <0.00001 | Random |
| Togoz 2013 [38] | 1.11 (1.07-1.16) | <0.00001 | 75 | <0.00001 | Random |
| Combined | 1.12 (1.07-1.16) | <0.00001 | 76 | <0.00001 | Random |
| **NLR and poor outcome** |  |  |  |  |  |
| Semerano 2020 [12] | 1.36 (1.21-1.53) | <0.00001 | 81 | <0.00001 | Random |
| Ying 2020 (OA) [14] | 1.32 (1.18-1.48) | <0.00001 | 83 | <0.00001 | Random |
| Ying 2020 (24H) [14] | 1.28 (1.15-1.43) | <0.00001 | 83 | <0.00001 | Random |
| Ying 2020 (7d) [14] | 1.26 (1.13-1.40) | <0.00001 | 81 | <0.00001 | Random |
| Cao 2020 [16] | 1.25 (1.12-1.39) | <0.00001 | 81 | <0.00001 | Random |
| Wang 2019 [17] | 1.38 (1.21-1.57) | <0.00001 | 82 | <0.00001 | Random |
| Semerano 2019 (24H) [19] | 1.26 (1.13-1.40) | <0.00001 | 81 | <0.00001 | Random |
| Semerano 2019 (OA) [19] | 1.29 (1.15-1.44) | <0.00001 | 83 | <0.00001 | Random |
| Lim 2019 [23] | 1.28 (1.15-1.43) | <0.00001 | 83 | <0.00001 | Random |
| Shi 2018 [10] | 1.30 (1.16-1.45) | <0.00001 | 83 | <0.00001 | Random |
| Duan 2018 [24] | 1.28 (1.15-1.43) | <0.00001 | 83 | <0.00001 | Random |
| Goyal 2018 [25] | 1.37 (1.20-1.57) | <0.00001 | 83 | <0.00001 | Random |
| Malhotra 2018 [26] | 1.27 (1.14-1.42) | <0.00001 | 82 | <0.00001 | Random |
| Wang 2018 [28] | 1.36 (1.19-1.54) | <0.00001 | 83 | <0.00001 | Random |
| Yu 2018 [29] | 1.30 (1.16-1.45) | <0.00001 | 83 | <0.00001 | Random |
| Xue 2017 [30] | 1.28 (1.14-1.43) | <0.00001 | 82 | <0.00001 | Random |
| Qun 2017 [31] | 1.25 (1.12-1.39) | <0.00001 | 81 | <0.00001 | Random |
| Brooks 2014 [36] | 1.28 (1.15-1.43) | <0.00001 | 82 | <0.00001 | Random |
| Combined | 1.29 (1.16-1.44) | <0.00001 | 82 | <0.00001 | Random |
| **NLR and sICH/HT** |  |  |  |  |  |
| Semerano 2020 [12] | 1.15 (1.07-1.24) | 0.0002 | 69 | 0.0002 | Random |
| Zhang 2020 (a) [13] | 1.16 (1.08-1.25) | 0.0001 | 72 | <0.0001 | Random |
| Ying 2020 (OA) [14] | 1.15 (1.08-1.24) | <0.0001 | 72 | <0.0001 | Random |
| Ying 2020 (24H) [14] | 1.14 (1.07-1.22) | <0.0001 | 69 | <0.0001 | Random |
| Świtońska 2020 [15] | 1.14 (1.07-1.22) | 0.0002 | 70 | 0.0002 | Random |
| Wang 2019 [17] | 1.17 (1.09-1.26) | <0.00001 | 58 | <0.0001 | Random |
| Semerano 2019 (OA) [19] | 1.15 (1.07-1.23) | <0.0001 | 71 | <0.0001 | Random |
| Semerano 2019 (24H) [19] | 1.16 (1.08-1.25) | <0.0001 | 71 | <0.0001 | Random |
| Duan 2018 [24] | 1.14 (1.07-1.22) | <0.0001 | 69 | 0.0002 | Random |
| Goyal 2018 [25] | 1.17 (1.08-1.26) | 0.0001 | 72 | <0.0001 | Random |
| Pikijia 2018 [27] | 1.17 (1.08-1.26) | 0.0001 | 72 | <0.0001 | Random |
| Guo 2016 [34] | 1.16 (1.07-1.25) | 0.0002 | 71 | <0.0001 | Random |
| Maestrini 2015 [35] | 1.13 (1.07-1.19) | <0.0001 | 56 | 0.009 | Random |
| Combined | 1.15 (1.08-1.23) | <0.0001 | 69 | <0.0001 | Random |
| AIS, acute ischemic stroke, NLR, neutrophil-to-lymphocyte ratio, sICH, spontaneous intracerebral hemorrhage, OA, On admission, OR, Odds ratio, CI, confidence interval, DFS, disease-free survival, OS, overall survival. | | | | | |
