# Supplementary material for: Prognostic Value of Neutrophil-to-Lymphocyte Ratio in Stroke: A Systematic Review and Meta-Analysis
Source: Front Neurol. 2021 Sep 24;12:686983. doi: 10.3389/fneur.2021.686983 (PMC8497704; doi:10.3389/fneur.2021.686983)
Supplement: Supplementary Table 3 — Sensitivity analyses for NLR in AHS patients. AHS, acute hemorrhagic stroke, mRS, modifie. [file Table_3.DOCX]

| **Sensitivity analysis for NLR in AHS patients.** | | | | | |
| --- | --- | --- | --- | --- | --- |
| Study omitted | OR (95% CI) | *P* value | Heterogeneity | | Effect model |
|  |  |  | I^2^ (%) | *P*_H_ value |  |
| **NLR and** **mortality** |  |  |  |  |  |
| Qi 2018 [39] | 1.37 (1.06-1.78) | 0.02 | 93 | <0.00001 | Random |
| Lattanzi 2018 [41] | 1.18 (1.05-1.33) | 0.007 | 92 | <0.00001 | Random |
| Tao 2017 (24H) [43] | 1.17 (1.05-1.30) | 0.004 | 91 | <0.00001 | Random |
| Sun 2017 [44] | 1.24 (1.10-1.39) | 0.0005 | 93 | <0.00001 | Random |
| Giede-Jeppe 2017 [45] | 1.38 (1.10-1.74) | 0.006 | 91 | <0.00001 | Random |
| Lattanzi 2016 [47] | 1.25 (1.09-1.43) | 0.001 | 93 | <0.00001 | Random |
| Wang 2016 [48] | 1.27 (1.12-1.44) | 0.0002 | 93 | <0.00001 | Random |
| Tao 2016 [49] | 1.14 (1.02-1.27) | 0.02 | 90 | <0.00001 | Random |
| Combined | 1.23 (1.09-1.39) | 0.001 | 93 | <0.00001 | Random |
| **NLR and poor outcome** |  |  |  |  |  |
| Qin 2019 [39] | 1.11 (1.03-1.25) | 0.008 | 94 | <0.00001 | Random |
| Giede-Jeppe 2019 [40] | 1.17 (1.05-1.31) | 0.006 | 91 | <0.00001 | Random |
| Qi 2018 [42] | 1.10 (1.01-1.19) | 0.003 | 87 | <0.00001 | Random |
| Tao 2017 [43] | 1.10 (1.02-1.18) | 0.02 | 93 | <0.00001 | Random |
| Sun 2017 [44] | 1.11 (1.03-1.20) | 0.009 | 94 | <0.00001 | Random |
| Giede-Jeppe 2017 [45] | 1.16 (1.06-1.28) | 0.002 | 94 | <0.00001 | Random |
| Lattanzi 2017 [46] | 1.06 (0.99-1.14) | 0.09 | 92 | <0.00001 | Random |
| Combined | 1.11 (1.03-1.20) | 0.007 | 93 | <0.00001 | Random |
| AHS, acute hemorrhagic stroke, mRS, modified Rankin Scale, NLR, neutrophil-to-lymphocyte ratio. | | | | | |
